# *Streptococcus pneumoniae* outbreaks and implications for transmission and control: a systematic review

**DOI:** 10.1186/s41479-018-0055-4

**Published:** 2018-11-05

**Authors:** Paul N. Zivich, John D. Grabenstein, Sylvia I. Becker-Dreps, David J. Weber

**Affiliations:** 1 Department of Epidemiology, Gillings School of Global Public Health, University of North Carolina Chapel Hill, Chapel Hill, NC USA; 2 Merck Research Laboratories, Merck & Co., Inc., Kenilworth, NJ USA; 3 Department of Family Medicine, University of North Carolina Chapel Hill, Chapel Hill, NC USA; 4 Division of Infectious Diseases, Department of Medicine, University of North Carolina Chapel Hill, Chapel Hill, NC USA

**Keywords:** *Streptococcus pneumoniae*, Pneumococcus, Outbreaks, Transmission, Epidemic, Cluster, Pneumococcal vaccine

## Abstract

**Background:**

*Streptococcus pneumoniae* is capable of causing multiple infectious syndromes and occasionally causes outbreaks. The objective of this review is to update prior outbreak reviews, identify control measures, and comment on transmission.

**Methods:**

We conducted a review of published *S. pneumoniae* outbreaks, defined as at least two linked cases of *S. pneumoniae*.

**Results:**

A total of 98 articles (86 respiratory; 8 conjunctivitis; 2 otitis media; 1 surgical site; 1 multiple), detailing 94 unique outbreaks occurring between 1916 to 2017 were identified. Reported serotypes included 1, 2, 3, 4, 5, 7F, 8, 12F, 14, 20, and 23F, and serogroups 6, 9, 15, 19, 22. The median attack rate for pneumococcal outbreaks was 7.0% (Interquartile range: 2.4%, 13%). The median case-fatality ratio was 12.9% (interquartile range: 0%, 29.2%). Age groups most affected by outbreaks were older adults (60.3%) and young adults (34.2%). Outbreaks occurred in crowded settings, such as universities/schools/daycares, military barracks, hospital wards, and long-term care facilities. Of outbreaks that assessed vaccination coverage, low initial vaccination or revaccination coverage was common. Most (73.1%) of reported outbreaks reported non-susceptibility to at least one antibiotic, with non-susceptibility to penicillin (56.0%) and erythromycin (52.6%) being common. Evidence suggests transmission in outbreaks can occur through multiple modes, including carriers, infected individuals, or medical devices. Several cases developed disease shortly after exposure (< 72 h). Respiratory outbreaks used infection prevention (55.6%), prophylactic vaccination (63.5%), and prophylactic antibiotics (50.5%) to prevent future cases. PPSV23 covered all reported outbreak serotypes. PCV13 covered 10 of 16 serotypes. For conjunctival outbreaks, only infection prevention strategies were used.

**Conclusions:**

To prevent the initial occurrence of respiratory outbreaks, vaccination and revaccination is likely the best preventive measure. Once an outbreak occurs, vaccination and infection-prevention strategies should be utilized. Antibiotic prophylaxis may be considered for high-risk exposed individuals, but development of antibiotic resistance during outbreaks has been reported. The short period between initial exposure and development of disease indicates that pneumococcal colonization is not a prerequisite for pneumococcal respiratory infection.

**Electronic supplementary material:**

The online version of this article (10.1186/s41479-018-0055-4) contains supplementary material, which is available to authorized users.

## Background

Discovered in 1881 independently by Louis Pasteur and George Sternberg [1], *Streptococcus pneumoniae* is a Gram-positive bacterial pathogen that may asymptomatically colonize the upper respiratory tract and is capable of causing infections including conjunctivitis, otitis media, lower respiratory tract infections, bacteremia, and meningitis [2]. Those at particularly high risk for invasive disease are young children, older adults, and persons with underlying comorbidities [3, 4]. Among United States (US) adults ≥50 years, it is estimated that *S. pneumoniae* causes ≥500,000 cases of pneumonia and ≥ 25,000 deaths each year [5]. Previous publications describing pneumococcal disease state that nasopharyngeal colonization is a prerequisite for disease [2, 6, 7]. Colonization is “the presence and multiplication of microorganisms without tissue invasion or damage” [8]. Conversely, infection involves tissue invasion.

The objective of this review was to summarize the publications on outbreaks and inform the understanding of *S. pneumoniae* transmission in these outbreaks. The most recent review of general pneumococcal outbreaks was conducted in 2010 [9]. Since then, the Advisory Committee on Immunization Practices (ACIP) has revised its recommendations to include the use of 13-valent pneumococcal conjugate vaccine (PCV13) in adults [10]. Our review represents an important update to previous reviews, includes additional pneumococcal disease manifestations, and has over double the number of included articles from the previous review. This review informs the understanding of *Streptococcus pneumoniae* outbreak serotypes, transmission, and effective control measures.

## Methods

A search of PubMed was conducted on July 18, 2017, for publications describing outbreaks of disease caused by *S. pneumoniae*. The following search terms were used: (“streptococcus pneumoniae” OR “pneumococcus”) AND (“outbreak” OR “epidemic”) with no date restrictions. Articles not available in the English language were excluded. All types of pneumococcal disease, year of outbreak, or location of outbreak were eligible for inclusion. To be considered an outbreak, at least one transmission event of pneumococcal disease had to occur. Pneumococcal carriage or surveillance studies were included if details of a pneumococcal outbreak were described. Each included article’s references and previous reviews [9, 11, 12] were screened for additional articles not identified.

The following information was extracted from publications. Case-patient ages were groups into five categories; toddler (0–2 years old), children (3–17), young adults (18–25), adults (26–49) and older adults (50+). *S. pneumoniae* were considered antibiotic susceptible or non-susceptible, where non-susceptible refers to intermediate or resistant. Specific antibiotic susceptibility information was extracted for penicillin, cefotaxime, erythromycin, tetracycline, levofloxacin, and vancomycin. The three general control measures considered were antibiotic prophylaxis, prophylactic vaccination, and infection prevention (i.e., hand-hygiene, isolation of cases, isolation of carriers, social distancing). Outbreak settings were categorized as occurring in hospitals, military, long term care facilities (LTCF), daycares, schools, jails, or workplaces. Settings falling outside these categories were grouped as “community” outbreaks. Pneumococcal lower respiratory tract infections were divided into three eras; pre-vaccine (pre-1977), pneumococcal polysaccharide vaccine (PPSV) only (1977–1999), and PPSV and PCV vaccines (2000–2017).

## Results

**Fig. 1:**
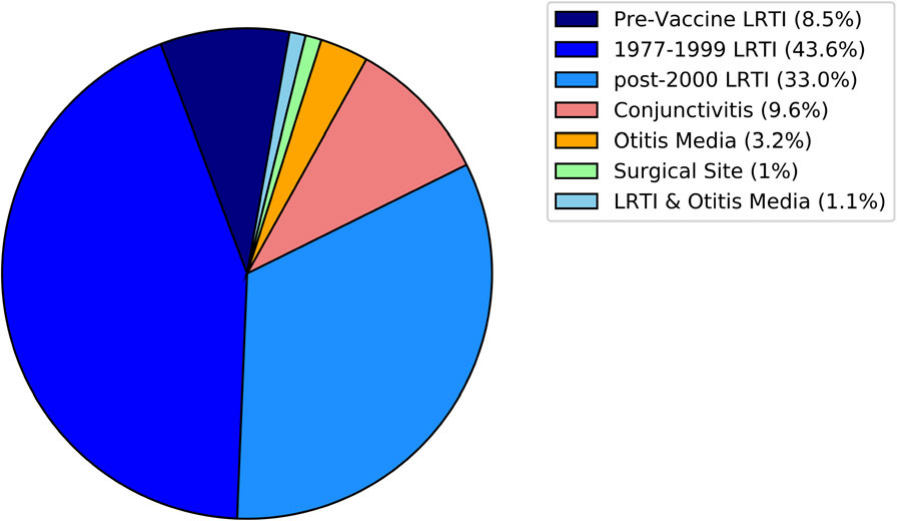
Reported *Streptococcus pneumoniae* outbreaks by anatomical site. LRTI: Lower respiratory tract infection. LRTI was divided into three eras; pre-vaccine (pre-1977), pneumococcal polysaccharide vaccine only (1977–1999), and pneumococcal polysaccharide and conjugate vaccines (2000–2017)

The search identified 629 potential articles. After screening, 83 articles were identified as meeting the inclusion criteria. From references of included articles and other reviews an additional 15 articles were identified. A total of 98 publications detailing 94 unique *S. pneumoniae* outbreaks were identified (Table 1, Additional file 1: Figure S1). Thirteen reports were published from 1916 to 1946, and the remainder were published after 1980. Unique outbreaks by disease syndrome were as follows; 80 lower respiratory tract infection [12–97], 9 conjunctivitis [98–105], 3 otitis media [106, 107], 1 surgical site infection [108], and 1 lower respiratory tract infection and otitis media [109] (Fig. 1).

**Table 1:** Characteristics of included pneumococcal publications

Author	Year published	Country	Type	Setting	Age Categories	Linkage	Serotype	Number colonized (%)	Number infected (%)	Case-Fatality Ratio
McCrae T	1916	Canada	Respiratory	Community						
Miller JL	1918	United States	Respiratory	Military					346	0.325
Schroder MC	1930	United States	Respiratory	School	Children		5		150	
Smillie WG	1936	United States	Respiratory	Hospital			2	21	17	0.471
Tilghman RC	1936	United States	Respiratory	Community	Toddler, Children, Young Adult, Adult, Older Adult		Variety			
Gilman BB	1938	United States	Multiple	Community		Neufeld	1		35 (7.0%)	0.114
Smillie WG	1938	United States	Respiratory	Hospital			1	(10.0%)	110	
Mackenzie GM	1940	United States	Respiratory	Community		Neufeld	1	43 (18.0%)	4 (1.6%)	
Hodges RG*	1946	United States	Respiratory	Military		Neufeld	Variety		1644	0
Hodges RG*	1946	United States	Respiratory	Military		Neufeld	Variety		1644	0
Hodges RG*	1946	United States	Respiratory	Military		Neufeld	Variety		1644	0
Hodges RG*	1946	United States	Respiratory	Military		Neufeld	Variety		1644	0
Hodges RG*	1946	United States	Respiratory	Military		Neufeld	Variety		1644	0
DeMaria A	1980	United States	Respiratory	Community		Quelleng	1	10 (10%)	39	
Shayegani M	1982	United States	Conjunctivis	Community			Nontypeable		1567	0
Shayegani M	1983	United States	Conjunctivis	Military			Nontypeable		80	0
Fenton PA	1983	United Kingdom	Respiratory	Community			1			
Shayegani M	1984	United States	Conjunctivis	Community	Adult, Older Adult		Nontypeable		1189	0
Davies AJ	1984	United Kingdom	Respiratory	Hospital		Immunoelectrophoresis	9		4	
Berk SL	1985	United States	Respiratory	Hospital	Older Adult	Quelleng	8	0 (0%)	4 (18.0%)	0.500
Collingham KE	1985	United States	Respiratory	Community	Older Adult		12		2	0.500
Mehtar S	1986	United Kingdom	Respiratory	Hospital	Toddler		6		3	0.333
Davies AJ	1987	United Kingdom	Respiratory	Hospital	Older Adult	Quelleng	3		8	0.250
Gould FK	1987	United Kingdom	Respiratory	Hospital	Older Adult		23		6	
Moore EP	1988	United Kingdom	Respiratory	Hospital	Older Adult		23		4	0.250
CDC*	1989	United States	Respiratory	Jail	Young Adult, Adult	Quelleng	12F	11 (7.0%)	17 (0.5%)	0.043
Rauch AM	1990	United States	Respiratory	Daycare	Toddler		14	10 (9.3%)	2 (2.4%)	0
Bain M	1990	United Kingdom	Respiratory	Hospital	Older Adult	Quelleng	4		4	
Mercat A	1991	France	Respiratory	Community	Young Adult, Adult, Older Adult	Quelleng	1	1 (2.0%)	39	0.040
Cartmill TDI	1992	United Kingdom	Respiratory	Hospital	Older Adult		6	1	4	0.250
Dawson S	1992	United Kingdom	Respiratory	Hospital	Older Adult		6	1	5	
PHLS	1992	United Kingdom	Respiratory	Hospital			9		7	0.143
Quick RE	1993	United States	Respiratory	Long-term care facility	Older Adult	Quelleng	9 V	2 (3.0%)	7 (7.4%)	0.710
Gratten M	1993	Australia	Respiratory	Community	Young Adult, Adult, Older Adult	Antisera	1	13 (17.3%)	18	
Denton M	1993	United Kingdom	Respiratory	Hospital	Older Adult		14		8	0.125
Hoge CW*	1994	United States	Respiratory	Jail	Young Adult, Adult	Quelleng	12F	11 (7.0%)	46 (0.5%)	0.043
Cherian T	1994	United States	Respiratory	Daycare	Toddler	Ribotyping	12F	6 (100%)	4 (66.7%)	0
Millar MR	1994	United Kingdom	Respiratory	Hospital	Older Adult		9	0 (0%)	10 (5.7%)	
Mandigers CMPW	1994	Netherlands	Respiratory	Hospital	Older Adult	Quelleng	9		18	0.556
Nims L	1994	United States	Respiratory	Daycare	Toddler	PCR	19	3	14	0.500
Raymond J	1995	France	Respiratory	Hospital	Toddler	RAPD	23F		2	
Ertugrul N	1997	United States	Conjunctivis	Military		PFGE, PCR	Nontypeable		561	0
Marton A	1997	Hungary	Otitis Media	Hospital	Toddler			0	6	
Marton A	1997	Hungary	Otitis Media	Hospital	Toddler				3	
Gillespie SH	1997	United Kingdom	Respiratory	Hospital	Older Adult	RFLP	9	3	9	0.444
CDC*	1997	United States	Respiratory	Long-term care facility	Older Adult	PCR	14		10 (14.9%)	0.200
CDC*	1997	United States	Respiratory	Long-term care facility	Older Adult	PFGE	23F	17 (23.0%)	11 (13.0%)	0.270
CDC	1997	United States	Respiratory	Long-term care facility	Older Adult	PCR	4		14 (11.7%)	0.290
Musher DM	1997	United States	Respiratory	Military	Young Adult		1		128 (3.2%)	
Musher DM	1997	United States	Respiratory	Military	Young Adult		7F / 8	44 (28.4%)	14 (6.4%)	
Fiore AE*	1998	United States	Respiratory	Long-term care facility	Older Adult	PCR	14		10 (14.9%)	0.200
Nuorti JP*	1998	United States	Respiratory	Long-term care facility	Older Adult	PFGE	23F	17 (23.0%)	11 (13.0%)	0.27
Sheppard DC	1998	United States	Respiratory	Long-term care facility	Older Adult	PFGE	14	0 (0%)	15 (12.5%)	
Razzaq N	1998	United Kingdom	Respiratory	Community	Toddler, Older Adult		12F		2	
Craig AS	1999	United States	Respiratory	Daycare	Toddler	PFGE	14	15 (19%)	3 (3.4%)	0
de Galan BE	1999	Netherlands	Respiratory	Hospital	Older Adult	Quelleng, RFEL	15		36	0.297
Kellner JD	1999	Canada	Respiratory	Community	Adult, Older Adult	PFGE			6	0.167
Leggiadro RJ	1999	United States	Respiratory	Long-term care facility	Older Adult			4	3	
Gleich S	2000	United States	Respiratory	Long-term care facility	Older Adult	PFGE	4		11 (5.5%)	
Dagan R	2000	Israel	Respiratory	Community		Ribotyping, PCR	1	31 (4.8%)	5	
Nakashima T	2001	Japan	Otitis Media	Daycare	Children	Antisera, RAPD	19, 23, 6		7	
CDC*	2001	United States	Respiratory	Long-term care facility	Older Adult	PFGE	14		9	0.444
Weiss K	2001	Canada	Respiratory	Hospital	Older Adult	PFGE	23F		23	0.087
CDC	2002	United States	Conjunctivis	School	Children, Young Adult, Adult	PFGE	Nontypeable		144	
Melamed R	2002	Israel	Respiratory	Hospital	Toddler	RAPD	5	0	3	
Martin M	2003	United States	Conjunctivis	Community	Young Adult	PFGE, MLST	Nontypeable	20 (8.1%)	698 (13.8%)	
Tan CG*	2003	United States	Respiratory	Long-term care facility	Older Adult	PFGE	14		9	0.444
Crum NF	2003	United States	Respiratory	Military	Young Adult	Quelleng, Latex Agglutination	9 V / 4	13 (11.0%)	52 (1.5%)	0
Subramanian D	2003	United Kingdom	Respiratory	Hospital		PFGE	9 V	3 (5.5%)	9	
Sanchez JL	2003	United States	Respiratory	Military	Young Adult	PCR	3, 6, 9, 14, 20, 22, 23	30 (13.6%)	30 (12.1%)	
Crum NF	2004	United States	Conjunctivis	Military	Young Adult, Adult	MLST	Nontypeable	15 (9.9%)	80 (2.3%)	0
Banerjee A	2005	India	Respiratory	Military		RAPD				0
CDC	2005	United States	Respiratory	Hospital	Adult	PFGE	23F, 3	6 (9.0%)	7	0.290
CDC*	2005	United States	Respiratory	Community	Toddler, Children, Young Adult, Adult, Older Adult	PFGE, MLST, MLBT	12F	46 (2.4%)	14 (0.1%)	
Birtles A	2005	United Kingdom	Respiratory	Community	Adult	MLST	8		2	1
Buck JM	2006	United States	Conjunctivis	Community	Toddler, Children, Young Adult, Adult, Older Adult	PFGE, MLST	Nontypeable		735	0
Hennick M	2006	Canada	Conjunctivis	Community	Toddler, Children, Young Adult, Adult, Older Adult	AFLP	Nontypeable		47	
Hansmann Y	2006	France	Respiratory	Long-term care facility	Older Adult	Antisera, Urine	4	1 (1.2%)	11 (11.7%)	0.273
Singh PMP	2006	India	Respiratory	Military	Young Adult				316	0
Cashman P	2007	Australia	Respiratory	School	Children		1		25	
Sheppard CL	2008	United Kingdom	Respiratory	Community	Children, Young Adult, Adult	PCR, MLST	1		11	0.182
Romney MG	2008	Canada	Respiratory	Community	Toddler, Children, Young Adult, Adult, Older Adult	Latex Agglutination	5		137	0.080
Zegans ME	2009	United States	Conjunctivis	Community	Young Adult	PFGE, MLST	Nontypeable	20 (8.1%)	698 (13.8%)	
Vainio A	2009	Finland	Respiratory	Military		MLST	7F	9 (20.9%)	5 (12.0%)	0
Gupta A	2009	United Kingdom	Respiratory	School	Children	ELISA, Urine	1	1 (1.2%)	5	
Mehiri-Zghal E	2010	Tunisia	Respiratory	Jail		PFGE	1	9 (45.0%)	150 (3.8%)	
Balicer RD	2010	Israel	Respiratory	Military	Young Adult	PFGE, MLST	5	35 (24.1%)	34	0
Pichon B	2010	United Kingdom	Respiratory	Community		MLST	5		8	
Dawood FS	2011	United States	Respiratory	Military	Young Adult	Quelleng	7F	16 (4.3%)	74 (0.3%)	0.027
Vanderkooi OG	2011	Canada	Respiratory	Community	Children, Young Adult, Adult, Older Adult	Quelleng, MLST	5, 8		207	
Skoczynska A	2012	Poland	Respiratory	Hospital	Older Adult	PFGE, MLST	14		6	
Fleming-Dutra K	2012	United States	Respiratory	Hospital	Children, Young Adult	PCR, MLST	15A	6	11	
Guillet M	2012	France	Surgical Site	Hospital			3	1	4	
Zulz T*	2013	United States	Respiratory	Community	Toddler, Children, Young Adult, Adult, Older Adult	PFGE, MLST, MLBT	12F	46 (2.4%)	14 (0.1%)	
CDC	2013	United States	Respiratory	Long-term care facility	Adult, Older Adult		3		7 (50.0%)	0.430
Kuroki T	2014	Japan	Respiratory	Hospital	Older Adult	PFGE, MLST	3		16 (83.9%)	0.063
Ben-David D	2014	Israel	Respiratory	Hospital	Young Adult, Older Adult	PFGE, MLST	19F, 23F	21 (20.2%)	66	
Schillberg E	2014	Canada	Respiratory	Community	Children, Young Adult, Adult, Older Adult	Quelleng, PFGE, MLST, MLVA	12F		32	
Suryam V	2015	India	Respiratory	Military					52	0
Thomas HL	2015	United Kingdom	Respiratory	Long-term care facility	Older Adult	Urinary, MLST	8		15 (65.0%)	0.133
Kunwar R	2015	India	Respiratory	Military	Young Adult				58 (1.1%)	0
Sheppard CL	2016	United Kingdom	Respiratory	Hospital	Older Adult	Urinary	6C		13	0.231
Ewing J	2017	United Kingdom	Respiratory	Workplace	Young Adult, Adult, Older Adult	WGS, MLST	4		25	0
Jauneikaite E	2017	United Kingdom	Respiratory	Hospital	Older Adult	WGS, MLST	9 V		4	

*PCR* polymerase chain reaction, *RAPD* random amplified polymorphic DNA, *PFGE* pulse-field gel electrophoresis, *RFLP* restriction fragment length polymorphism, *RFEL* restriction fragment end labeling, *MLST* multilocus sequence type, *AFLP* amplified fragment length polymorphism, *ELISA* enzyme-linked immunosorbent assay, *MLBT* multilocus boxB sequence typing, *MLVA* multiple loci variable-number tandem repeat analysis, *WGS* whole genome sequencing

Age categories are defined as follows; toddler (0–2 years old), children (3–17), young adult (18–25), adult (26–49), and older adult (50+)

Settings falling outside the other indicated categories were considered as “Community” settings. These included transmission among families, homeless shelter outbreaks, outbreaks in socially disadvantaged groups, and transmission occurring generally within geographical regions

*Outbreaks that were described in multiple publications. See supplement data set containing unique identifiers for each outbreak report

**Fig. 2:**
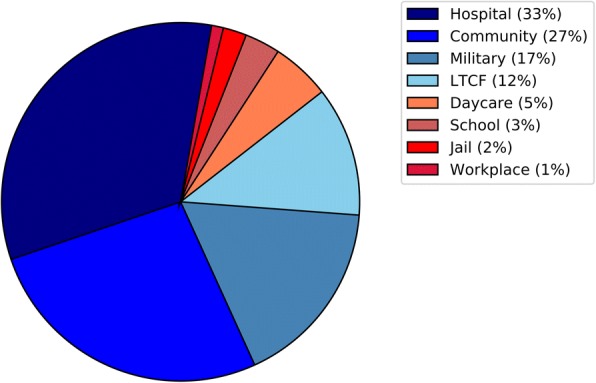
*Streptococcus pneumoniae* outbreaks by setting. LTCF: Long-term care facility. Graphic includes outbreaks from all anatomical sites (94 outbreaks)

A majority of reported outbreaks occurred in hospitals (33.0%), community (26.6%), or military buildings (17.0%) (Fig. 2). The most common age categories for case-patients in outbreaks (*n* = 73) were older adults (60.3%), young adults (34.2%) and adults (28.8%). Case-patients were less commonly toddlers (20.5%) or children (19.2%). Most reported outbreaks were reported in the US (43.6%), the United Kingdom (24.5%), or Canada (7.4%). France, India, and Israel each reported four outbreaks (4.3%); Japan, Australia, Netherlands, and Hungary each reported two outbreaks (2.1%); and Tunisia, Poland, and Finland each reported one outbreak (1.1%).

Sixty-one outbreak investigations reported assessing *S. pneumoniae* strains by molecular typing. The most common methods used were pulse-field-gel-electrophoresis (PFGE) (23.2%), antisera methods (23.2%), and multi-locus-sequence-typing (MLST) (22.0%). Of outbreak reports published since 2007 (*n* = 18), MLST (40.6%) and PFGE (18.8%) were most commonly used. Of 52 outbreaks assessing antibiotic resistance, 73.1% of outbreaks reported some antibiotic non-susceptibility. Antibiotics chosen for susceptibility testing were inconsistent. Non-susceptibility to penicillin (28/50 outbreaks), erythromycin (20/38), and tetracycline (11/20) were reported. Fewer outbreaks reported non-susceptibility to cefotaxime (5/13) or levofloxacin (3/11). Non-susceptibility to vancomycin was not reported for any outbreak (*n* = 17).

### Disease types

#### Lower respiratory tract infection

A total of 81 unique reported outbreaks involved lower respiratory tract infection with pneumococcus, with 9 in the pre-vaccine era, 41 in the PPSV era and 31 in the PPSV/PCV era.

##### Pre-vaccine era

Within the pre-vaccine era, outbreaks occurred in community (4/9), military (2/9), hospital (2/9), and a school (1/9) settings. Interestingly, 3 of the outbreak reports mentioned concocting a vaccine from pneumococcal polysaccharides [16, 18, 20].

##### PPSV era

**Fig. 3:**
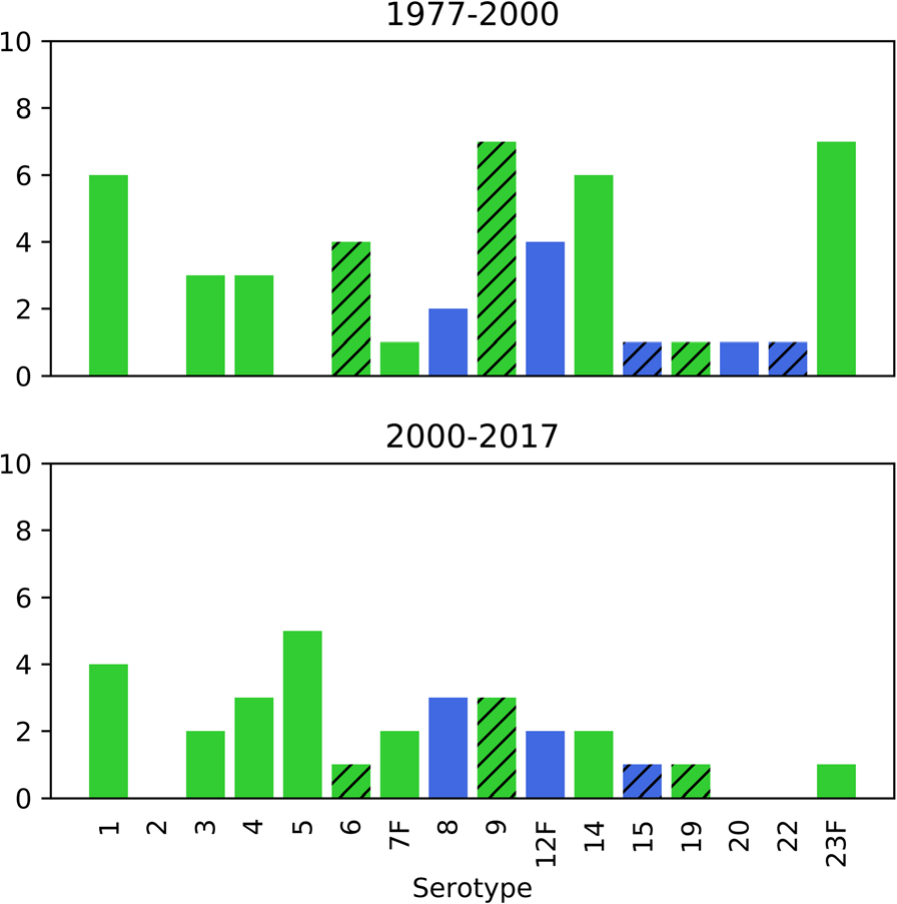
Pneumococcal lower respiratory tract infection outbreak serotypes and coverage by pneumococcal vaccines. Green: both the 13-valent pneumococcal conjugate vaccine (PCV13) and 23-valent pneumococcal polysaccharide vaccine (PPSV23) cover the indicated serotype. Blue: only PPSV23 covers the indicated serotype. Hatched bars indicate serogroups that have subtypes covered by the vaccines, but the specific serotype within the serogroup was not consistently reported across publications. The graph is subdivided by vaccine era; PPSV only (1977–1999) and PPSV/PCV (2000–2017)

During the PPSV era, reported outbreaks occurred in hospitals (43.9%), community (19.5%), LTCF (17.1%), daycares (9.8%), military (7.3%) or jail (2.4%) settings. Within hospital settings, outbreaks occurred in geriatric, pulmonary, oncology, maternity, and “AIDS-care” units. Community outbreaks included homeless shelter outbreaks, transmission between family members, and outbreaks occurring within socially disadvantaged groups. Of the 39 outbreaks that reported serotypes, the most common pneumococci were serogroup 9 (15.4%), serotype 1 (15.4%), serotype 23F (12.8%), and serotype 14 (12.8%) (Fig. 3). Of the 17 studies that reported colonization data, the median percent of colonized individuals was 9.3% (IQR: 3.0%, 19.0%). For 15 studies with a denominator, the median attack rate was 7.4% (IQR: 4.4%, 12.8%) with a median case-fatality ratio of 25.0% (IQR: 11.5%, 36.1%) from 24 studies. Twenty-six studies reported conducting testing for resistance to at least one antibiotic. Non-susceptibility was reported for the following antibiotics; penicillin (18/25), cefotaxime (5/9), erythromycin (9/19), tetracycline (8/11), levofloxacin (2/3), and other antibiotics (13/17). No vancomycin non-susceptibility was reported in 13 publications. Seven outbreaks reported sufficient information to calculate the vaccination coverage of the source population with the following coverages; 2% [110], 3% [55], two with 4% [52, 110], 7% [42], and 24% [70]. One study reported an unadjusted vaccine effectiveness (VE) of 0.87 (95% CI: -0.03, 0.98) for those who received PPSV before the outbreak [70]. For hospital outbreaks with reported control measures (11/18), infection-prevention practices alone (54.5%), vaccination alone (9.1%), infection-prevention and vaccination (18.2%), infection-prevention and prophylactic antibiotics (9.1%), and all three (9.1%) were used to mitigate outbreaks. Only two outbreaks (infection control alone [64], all three control measures [70]) reported control measures as unsuccessful. Both outbreaks described the development of antibiotic resistance over the course of the outbreak [64, 70]. LTCF reported infection-prevention and vaccination (2), infection-prevention and antibiotics (1), and all three (1) as control measures with cases discontinuing after implementation. Two of three daycares that used antibiotics alone reported failure of control measures to eradicate carriage of the outbreak strain. For outbreaks designated as within communities, a homeless men’s shelter controlled an outbreak successfully using vaccination. None of the other community outbreaks reported using control measures.

##### PPSV/PCV era

From 2000 to 2017, outbreaks were reported in hospitals (25.8%), military settings (25.8%), communities (22.6%), LTCF (12.9%), schools (6.5%), a workplace (3.2%), and a jail (3.2%). Hospital outbreaks occurred in geriatric, pulmonary, ear/nose/throat, and a pediatric psychiatry ward. Community outbreaks included a homeless shelter outbreak, transmission among children, and a socially disadvantaged group. Of outbreaks with recorded case-patient ages (*n* = 29), 55.2% were older adults, 48.3% were young adults, 37.9% were adults, 27.6% were children, and 10.3% were toddlers. Twenty-seven outbreaks reported serotypes, with serotype 5 (18.5%) and serotype 1 (14.8%) most commonly reported (Fig. 3). Of 10 outbreaks with a denominator for colonization, the median colonization percentage was 8.2% (IQR: 2.9%, 20.7%). The median attack rate was 7.7% (IQR: 1.2%, 40.5%) for the ten outbreaks that provided attack rates. The case-fatality ratio was 4.5% (IQR: 0%, 21.9%) for 18 reports. Seventeen studies reported testing for antibiotic resistance with 64.7% reporting resistance to at least one antibiotic. Reported antibiotic non-susceptibility included; penicillin (5/16), erythromycin (5/11), tetracycline (2/5), levofloxacin (1/4), and other antibiotics (5/9). No non-susceptibility was reported for cefotaxime (*n* = 3) and vancomycin (*n* = 2). Twelve studies assessed whether case-patients had ever received either pneumococcal vaccine before the outbreak [62, 66, 67, 71, 73, 74, 82, 88, 89, 91, 93, 97]. Of the studies that provided enough information to calculate vaccination coverage of the source population of cases, two reported 0% coverage [66, 88], one reported 7% [89], and one reported 57% [93]. At least one vaccine failure was reported for 6 studies [71, 74, 88, 91, 93, 97]. Two reports described one case-patient vaccine failure of a vaccine received within five years of the outbreak [71, 74]. Two studies reported PPSV VE among older adults; 1.00 (95% CI: 0.30, 1.00) [63] and − 0.41 (95% CI: -2.33, 0.40) [93]. The poor VE and the outbreak occurring despite 57% vaccination coverage was partially attributed to “waning immunity” by the authors, since all case-patients received the vaccine more than 7 years prior to the outbreak [93]. Of hospital outbreaks with reported control measures (6/8), the following measures were used; infection-prevention alone (16.7%), vaccination alone (16.7%), infection-prevention and prophylactic antibiotics (50.0%), and vaccination and prophylactic antibiotics (16.7%). None of the six outbreaks reported the control measures failing to control the outbreak. Military outbreaks were effectively controlled by antibiotics alone (1), infection control alone (1), antibiotics and vaccination (3), infection-prevention and antibiotics (3) or all three (1). All five LTCF outbreaks were controlled with vaccination paired with infection control (2), antibiotics (2), or both (1). Community outbreaks reporting control measures (5/7), all used vaccination alone. All outbreaks except one were reported as being successfully controlled.

### Conjunctivitis

Eight publications describing nine conjunctivitis outbreaks have been published since 1982. All of these outbreaks are attributed to non-typeable strains. Two pneumococcal outbreaks were MLST sequence type 448 [101, 104]. Interestingly, this pneumococcal strain, identified in the 2002 Dartmouth and 2003 Minnesota outbreaks, was related to a strain isolated in 1980’s outbreaks in New York, California, and Illinois [98, 99, 101, 104]. Noteworthy is the development of non-susceptibility to penicillin, erythromycin, and tetracycline between 1980 to 2003 in this strain. Four of six outbreaks reported non-susceptibility to at least one antibiotic [98, 100, 103, 104]. Non-susceptibility was observed for erythromycin (4/6), penicillin (2/6) and tetracycline (1/4). Outbreaks occurred in the community (*N* = 6) or military settings (*N* = 3). Three of the community outbreaks were associated with universities, with two large outbreaks occurring in this setting. Furthermore, all outbreaks with reported ages (*n* = 5) included young adult case-patients. Outbreaks of conjunctivitis were generally larger than respiratory outbreaks (median: 561 cases; range 80, 735). There was no reported mortality associated with these outbreaks. Five outbreaks reported using infection-prevention to control outbreaks and led to a subsequent decline in cases [100, 101, 103–105]. However, for three of the four outbreaks related to schools, the decline occurred after school breaks [100, 101, 105], complicating the attribution of infection-prevention strategies as ending the outbreak.

### Otitis media

There were four reported otitis media outbreaks in three publications. The first was an otitis media outbreak occurring simultaneously with a pneumococcal lower respiratory tract infection outbreak in a US community in 1937 [109]. This study was unable to directly link the two manifestations of pneumococcal disease. Two outbreaks occurred in hospitals in Hungary during 1993–1994 and 1996 [106]. No carriers were reported among healthcare personnel and transmission was believed to occur between patients in the hospital since case-patients shared rooms. The other outbreak occurred in 1997 in a Japanese daycare center among seven children with serogroup 6 (*n* = 1), and serotypes 19 (*n* = 4), and 23F (*n* = 2) [107]. Otitis media occurred in at least one case-patient during two pneumococcal lower respiratory tract infection outbreaks [48, 61]. Non-susceptibility was reported for penicillin (3/3) and erythromycin (2/2). None of the publications reported instituting control measures.

### Surgical-site

One publication detailed four surgical site infections transmitted by a surgeon with nasopharyngeal carriage to four prostatic surgery patients [108]. Pneumococcal infection occurred at skin and soft tissue near the surgical site of case-patients. Transmission was attributed to the surgeon persistently wearing a poorly fitting mask.

### Transmission

In the reviewed outbreak articles, we found evidence for multiple modes of transmission. Aside from transmission attributable to nasopharyngeal carriers, there were pneumococcal lower respiratory tract infection outbreaks suggestive of device-associated transmission (infant resuscitation device [30]; inhaler [50, 97]), and infection transmission without any carriers detected [27, 28, 33, 39, 46, 65, 97]. While it is near impossible to ever fully rule out transient nasopharyngeal colonization as the source, these outbreaks found no evidence that carriers contributed to transmission. There is also evidence that droplet transmission occurs for *S. pneumoniae*. In a neonatal intensive care unit, transmission occurred between two neonates 2 meters apart who had no overlapping nursing staff, no contact between families, no carriage among family member, and infection of the transmitting neonate occurred before admission [65]. Studies also reported co-circulation of other viral [51, 76, 87, 91, 95] or bacterial respiratory tract pathogens [44, 59, 84, 87] preceding or during a pneumococcal outbreak. For conjunctivitis outbreaks, direct droplet, or indirect (i.e. environment or hand contamination) transmission may also have occurred, because having a roommate with conjunctivitis was associated with developing conjunctivitis [101]. In four outbreaks, time from exposure to infection in several outbreaks was less 72 hours for at least one case-patient [27, 28, 65, 97]. Serotype 1, 5, and 9 V exhibited short times between exposure and disease.

## Discussion

**Fig. 4:**
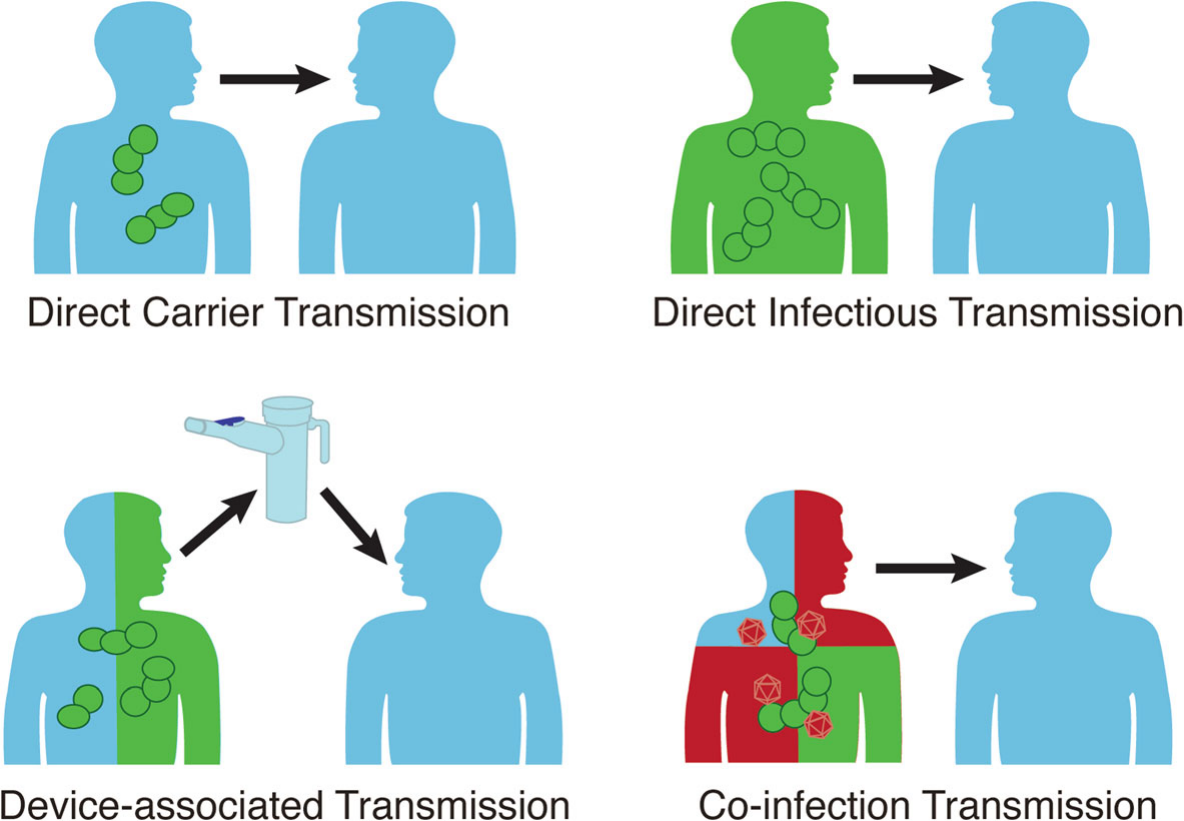
Modes of person-to-person transmission of *Streptococcus pneumoniae*

**Fig. 5:**
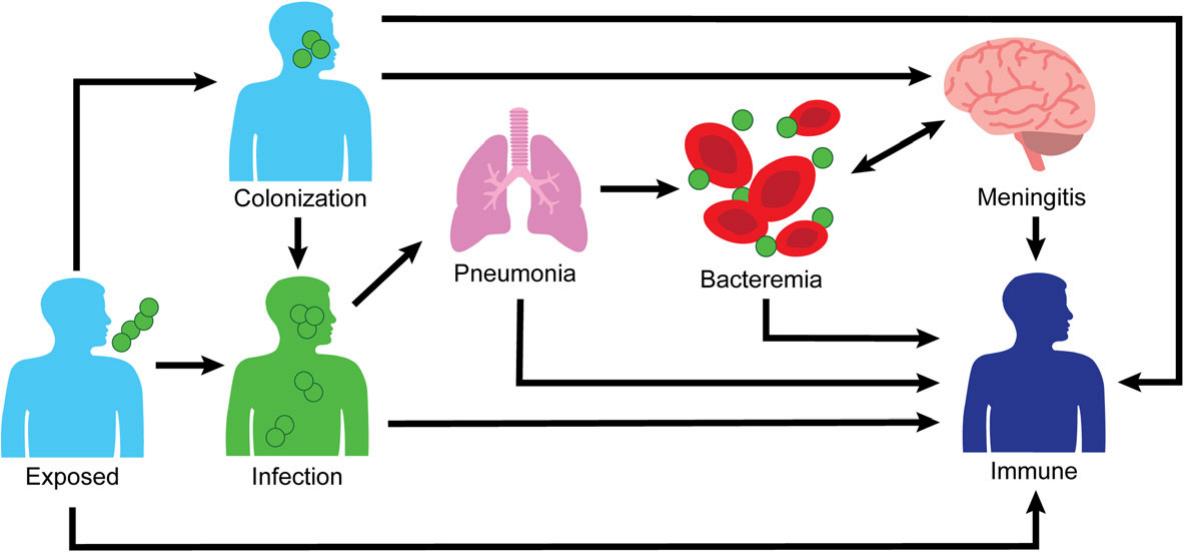
Simplified description of serious *Streptococcus pneumoniae* infections, with a focus on initial respiratory tract disease. Death, not represented in the figure, can occur at any illness stage with varying survival probability based on disease stage

In our review, we found multiple outbreaks attributable to *S. pneumoniae* reported in the first half of the 1900s. In the beginning of the antibiotic area, there were no publications regarding pneumococcal outbreaks. One explanation for this observation is that outbreaks went unreported or unrecognized due to widespread antibiotic use while antibiotics were exquisitely effective. After the 1980’s, outbreaks began being reported regularly, with most reporting non-susceptibility to at least one antibiotic. A majority of pneumococcal outbreaks are linked to lower respiratory tract infection but several large conjunctivitis outbreaks due to non-typeable strains have recently occurred. Most reported lower respiratory tract infection outbreaks have occurred in hospitals, perhaps due to observation bias. Conjunctivitis outbreaks have mostly occurred in community settings, specifically universities*.* Regarding transmission, this review supported the view of *S. pneumoniae* transmission as a complicated process occurring by multiple modes (Fig. 4), and droplet precautions may be warranted for symptomatic patients due to the evidence of droplet transmission occurring [65]. Recommendation for droplet precautions is in line with American Public Health Association for patients with antibiotic-resistant pneumococcus [111], but differs from US Centers for Disease Control and Prevention’s (CDC) and Red Book’s recommendation of standard precautions for pneumococcus cases [112, 113]. For infection progression in individuals, we reviewed several studies that development of pneumococcal disease occurred with 72 h of initial exposure to *S. pneumoniae* [27, 28, 65, 97]. Such a short time between exposure and infection suggests that colonization is not a prerequisite for pneumococcal disease, and infection can progress directly from initial exposure. Based on these observations, we propose a new conceptual model for pneumococcal lower respiratory tract infection progression within an individual (Fig. 5). After initial exposure, an individual may develop infection directly or become colonized. A colonized individual can either develop disease or develop immunity to the pneumococcal serotype. After this point, pneumococcal respiratory infection progresses as has been described previously [2].

Of the serotypes reported in pneumococcal lower respiratory tract infection outbreaks, the reported strains are considered high risk for serious disease manifestation [114]. Specifically, increased empyema/parapneumonic effusion (serotype 1), meningitis (serotypes 12, 23F), and fatality (serotypes 14, 23F). These strains have continued to be reported in the PPSV/PCV era. Outbreak strains in lower respiratory tract infection outbreaks are included within PCV13 but are covered more fully by PPSV23 (Fig. 3) [115]. Along with the observation that most outbreaks occurred where vaccination/revaccination rates were low suggests that effective vaccination programs play a key role in preventing outbreaks. Providing vaccination is particularly vital in highly susceptible populations, like individuals in LTCF. Primary adult pneumococcal vaccination is recommended for healthy adults 65 years or older, immunocompromised individuals, and those with certain chronic diseases [115, 116]. Providing a 5-year PPSV23 revaccination, if indicated per US CDC recommendations, should be considered to retain sufficient immunity [117–121]. In regards to conjunctivitis, vaccination likely has no role in prevention since these strains do not express capsules, the antigenic target of the current vaccines.

The nasopharyngeal carrier state is an important feature in transmission of *S. pneumoniae* strains both within households and across regions. There is recognition that carrier-attributed transmission is important among families, with children acting as a reservoir [122–125]. In a global view, large events offer opportunities for widespread dissemination of *S. pneumoniae* strains. Events like the Hajj, the annual Islamic pilgrimage to Mecca, can lead to acquisition of new *S. pneumoniae* strains in attendees [126]. While the colonization has been important in dissemination of *S. pneumoniae*, in outbreaks we found evidence of additional transmission from other sources. Furthermore, there is a need to explore the transmission dynamics of *S. pneumoniae* with other respiratory pathogens, and the role of the time-order of co-infections [127]. Pneumococcal infection severity has been observed to increase with influenza in murine models [128], and influenza and other respiratory viruses have been associated with increased pneumococcal colonization and infection [129, 130]. In outbreak settings, interventions targeted at preventing or treating co-infections has potential to interrupt transmission. The CDC provided an interim recommendation for the use of PPSV23 as an adjunctive intervention during the 2009 influenza pandemic [131]. Data supports the use of pneumococcal vaccine in future influenza pandemics [132]. Additionally, annual influenza vaccination has potential to mitigate pneumococcal risk [133]. However, interventions targeted specifically for pneumococcus are still required to prevent pneumococcal outbreaks, as evidenced by a pneumococcal outbreak occurring in a military barrack with comprehensive influenza vaccination coverage [82].

Compared to the US CDC’s Active Bacterial Core Surveillance 2015 report for *S. pneumoniae* [134], reported outbreaks are much more likely to involve non-susceptible pneumococcal strains. The difference is likely related to publication bias favoring non-susceptible outbreak strains. Multitudes of pneumococcal disease outbreaks probably occur but are undetected due to inadequate diagnostic methods or effective antibiotic treatments, and we likely only see a fraction of the full burden of pneumococcal disease. If antibiotic resistance increases in the future, recognized pneumococcal outbreaks may occur with increasing frequency. However, childhood pneumococcal vaccination programs have been associated with a decrease in antibiotic resistance for vaccine serotypes in both children and adults [135], and may provide a way to reduce antibiotic resistance.

In future pneumococcal outbreaks, efforts should be made to rapidly identify cases and carriers to isolate them. For case linkage, we recommend using molecular typing methods, such as whole-genome sequencing (WGS), PFGE, or MLST, rather than serotyping alone. WGS is preferred over PFGE/MLST, but when not possible PFGE/MLST should be used. Recent outbreak investigations have been moving in this direction. When an outbreak is recognized, prompt vaccination or revaccination is important, but due to the delay until immunity occurs, infection-prevention measures are imperative. There is evidence that *S. pneumoniae* may be transmitted via droplets, so appropriate infection prevention measures should be taken (i.e. droplet precautions). While use of prophylactic antibiotics have had success in controlling outbreaks, the risk of antibiotic resistance developing should be considered carefully. Antibiotic non-susceptibility has previously developed secondary to antibiotic prophylaxis [70]. Rather, it may be appropriate to limit antibiotic prophylaxis to exposed contacts who are at high-risk of disease. Use of prophylactic antibiotics should be evaluated in light of the outbreak size, the pace of new cases, existing antibiotic resistances, and other contextual features. In conjunctivitis outbreaks, prevention efforts should focus on infection-prevention, since vaccination confers no protection against this disease manifestation.

The major strength of our review involves fewer restrictions on inclusion, allowing a more expansive assessment compared to prior pneumococcal outbreak reviews. We updated prior reviews with more recently published outbreaks. Our review also explored features of transmission and infection dynamics in *S. pneumoniae*, which has not previously been commented on in prior outbreak reviews. Lastly, since we could not report every possible combination of variables that may be of interest to readers, we have provided a data file containing all of the information extracted from the articles (Additional file 2: Table S1).

There are several limitations to our review. While our search terms were general, it is possible that our review missed articles of interest. We attempted to minimize this by searching through the references of included articles and other review articles. Some of these further identified articles were in journals not indexed by PubMed and would not have been identified regardless of search terms. One article not identified by our search reported a serotype 5 outbreak among unaccompanied minors in the US during 2014 [136]. Our conclusions are consistent with the unidentified article and this article provides further evidence for co-infection transmission. Our search was limited to only including articles available in English, but only 8 non-English were identified as eligible via abstracts. Lastly, our review is limited to published outbreaks. However, our conclusions regarding transmission and infection progression remain valid, because only one example is needed to show this can occur.

## Conclusion

*S. pneumoniae* causes outbreaks of various clinical manifestations. There is sufficient evidence that *S. pneumoniae* colonization is not an obligate prerequisite for disease. To prevent the initial occurrence of outbreaks, maintaining high vaccination rates and revaccination per US CDC/ACIP recommendations is likely to be effective. Once an outbreak occurs, efforts should be directed to infection-prevention strategies, like droplet precautions, and vaccination. The usage of prophylactic antibiotics for exposed individuals may lead to development of antibiotic resistance, and is not currently recommended by the CDC. In scenarios of pneumococcal infection co-circulating with another pathogen, interventions targeted at the co-circulating infections may mitigate pneumococcal transmission. Interestingly, conjunctival pneumococcal outbreaks have been linked to bacteria that do not express a capsule and would therefore not be covered by the currently-licensed pneumococcal vaccines. Despite being discovered over 100 years ago, there is still much to uncover regarding *S. pneumoniae*.

## Additional files

Additional file 1: **Figure S1.** Article exclusion flow diagram. (PNG 22 kb)

Additional file 2: **Table S1.** Data extracted from reviewed articles. (XLSX 28 kb)

## References

[1] Watson DA, Musher DM, Jacobson JW, Verhoef J (1993). A brief history of the pneumococcus in biomedical research: a panoply of scientific discovery. Clin Infect Dis.

[2] Henriques-Normark B., Tuomanen E. I. (2013). The Pneumococcus: Epidemiology, Microbiology, and Pathogenesis. Cold Spring Harbor Perspectives in Medicine.

[3] Ortqvist A, Hedlund J, Kalin M (2005). Streptococcus pneumoniae: epidemiology, risk factors, and clinical features. Semin Respir Crit Care Med.

[4] Torres A, Blasi F, Dartois N, Akova M (2015). Which individuals are at increased risk of pneumococcal disease and why? Impact of COPD, asthma, smoking, diabetes, and/or chronic heart disease on community-acquired pneumonia and invasive pneumococcal disease. Thorax.

[5] Weycker D, Strutton D, Edelsberg J, Sato R, Jackson LA (2010). Clinical and economic burden of pneumococcal disease in older US adults. Vaccine.

[6] Tan TQ (2012). Pediatric invasive pneumococcal disease in the United States in the era of pneumococcal conjugate vaccines. Clin Microbiol Rev.

[7] Simell B, Auranen K, Kayhty H, Goldblatt D, Dagan R, O'Brien KL (2012). The fundamental link between pneumococcal carriage and disease. Expert Rev Vaccines.

[8] Mosby's. Mosby's medical dictionary. 7th edn. St. Louis: Elsevier; 2006.

[9] Ihekweazu C, Basarab M, Wilson D, Oliver I, Dance D, George R, Pebody R (2010). Outbreaks of serious pneumococcal disease in closed settings in the post-antibiotic era: a systematic review. J Inf Secur.

[10] Nuorti JP, Whitney CG (2010). Prevention of pneumococcal disease among infants and children - use of 13-valent pneumococcal conjugate vaccine and 23-valent pneumococcal polysaccharide vaccine - recommendations of the advisory committee on immunization practices (ACIP). MMWR Recomm Rep.

[11] Basarab M, Ihekweazu C, George R, Pebody R (2011). Effective management in clusters of pneumococcal disease: a systematic review. Lancet Infect Dis.

[12] Gleich S, Morad Y, Echague R, Miller JR, Kornblum J, Sampson JS, Butler JC (2000). Streptococcus pneumoniae serotype 4 outbreak in a home for the aged: report and review of recent outbreaks. Infect Control Hosp Epidemiol.

[13] McCrae T (1916). An epidemic of pneumococcus infection. Can Med Assoc J.

[14] Miller JL, Lusk FB (1918). Epidemic of streptococcus pneumonia and empyema at camp dodge, Iowa. J Am Med Assoc.

[15] Schroder MC, Cooper G (1930). An epidemic of colds, bronchitis and pneumonia due to type V pneumococci. J Infect Dis.

[16] Smillie WG (1936). A study of an outbreak of type ii pneumococcus pneumonia in the VETERANS' administration hospital at BEDFORD, MASSACHUSETTS1. Am J Epidemiol.

[17] Tilghman RC, Finland M (1936). PNEUMOCOCCIC infections in families. J Clin Investig.

[18] Smillie WG, Warnock GH, White HJ (1938). A study of a type I pneumococcus epidemic at the state Hospital at Worcester, mass. Am J Public Health Nations Health.

[19] Mackenzie G, McKee T, Tepperman J (1940). Epidemiology of an outbreak of pneumococcal pneumonia in a rural community. Trans Assoc Am Phys.

[20] Hodges RG, Macleod CM (1946). EPIDEMIC PNEUMOCOCCAL PNEUMONIAI. Description of the EPIDEMIC12. Am J Epidemiol.

[21] Hodges RG, Mac LC (1946). Epidemic pneumococcal pneumonia; the influence of population characteristics and environment. Am J Hyg.

[22] Hodges RG, Macleod CM, Bernhard WG (1946). EPIDEMIC PNEUMOCOCCAL PNEUMONIAIII. Pneumococcal carrier STUDIES12. Am J Epidemiol.

[23] Hodges RG, Macleod CM (1946). EPIDEMIC PNEUMOCOCCAL PNEUMONIAIV. The relationship of nonbacterial respiratory disease to pneumococcal PNEUMONIA12. Am J Epidemiol.

[24] Hodges RG, Mac LC (1946). Epidemic pneumococcal pneumonia; final consideration of the factors underlying the epidemic. Am J Hyg.

[25] DeMaria A, Browne K, Berk SL, Sherwood EJ, McCabe WR (1980). An outbreak of type 1 pneumococcal pneumonia in a men's shelter. Jama.

[26] Fenton PA, Spencer RC, Savill JS, Grover S (1983). Pneumococcal bacteraemia in mother and son. Br Med J (Clin Res Ed).

[27] Davies A J, Hawkey P M, Simpson R A, O'Connor K M (1984). Pneumococcal cross infection in hospital. BMJ.

[28] Berk SL, Gage KA, Holtsclaw-Berk SA, Smith JK (1985). Type 8 pneumococcal pneumonia: an outbreak on an oncology ward. South Med J.

[29] Collingham KE, Littlejohns PD, Anfilogoff N, Wiggins J (1985). Pneumococcal meningitis in a husband and wife. J Infect.

[30] Mehtar S, Drabu YJ, Vijeratnam S, Mayet F (1986). Cross infection with Streptococcus pneumoniae through a resuscitaire. Br Med J (Clin Res Ed).

[31] Davies AJ, Lockley MR (1987). A prospective survey of hospital cross-infection with Streptococcus pneumoniae. J Hosp Infect.

[32] Gould FK, Magee JG, Ingham HR (1987). A hospital outbreak of antibiotic-resistant Streptococcus pneumoniae. J Inf Secur.

[33] Moore EP, Williams EW (1988). Hospital transmission of multiply antibiotic-resistant Streptococcus pneumoniae. J Infect.

[34] Centers for Disease Control and Prevention. Outbreak of invasive pneumococcal disease in a jail--Texas, 1989. MMWR Morb Mortal Wkly Rep. 1989;38:733–734. 2509879

[35] Hoge CW, Reichler MR, Dominguez EA, Bremer JC, Mastro TD, Hendricks KA, Musher DM, Elliott JA, Facklam RR, Breiman RF (1994). An epidemic of pneumococcal disease in an overcrowded, inadequately ventilated jail. N Engl J Med.

[36] Rauch AM, O'Ryan M, Van R, Pickering LK (1990). Invasive disease due to multiply resistant streptococcus pneumoniae in a Houston, tex, day-care center. Am J Dis Child.

[37] Bain M, Ahmad N, Elder AT (1990). Pneumococcal cross-infection in hospitalized elderly patients. Br J Hosp Med.

[38] Mercat A, Nguyen J, Dautzenberg B (1991). An outbreak of pneumococcal pneumonia in two men's shelters. Chest.

[39] Cartmill TD, Panigrahi H (1992). Hospital outbreak of multiresistant Streptococcus pneumoniae. J Hosp Infect.

[40] Dawson S, Pallett A, Davidson A, Tuck A (1992). Outbreak of multiresistant pneumococci. J Hosp Infect.

[41] A nosocomial outbreak of Streptococcus pneumoniae infection. Commun Dis Rep CDR Wkly 1992, 2**:**29. 1285185

[42] Quick RE, Hoge CW, Hamilton DJ, Whitney CJ, Borges M, Kobayashi JM (1993). Underutilization of pneumococcal vaccine in nursing home in Washington state: report of a serotype-specific outbreak and a survey. Am J Med.

[43] Gratten M, Morey F, Dixon J, Manning K, Torzillo P, Matters R, Erlich J, Hanna J, Asche V, Riley I (1993). An outbreak of serotype 1 Streptococcus pneumoniae infection in Central Australia. Med J Aust.

[44] Denton M, Hawkey PM, Hoy CM, Porter C (1993). Co-existent cross-infection with Streptococcus pneumoniae and group B streptococci on an adult oncology unit. J Hosp Infect.

[45] Cherian T, Steinhoff MC, Harrison LH, Rohn D, McDougal LK, Dick J (1994). A cluster of invasive pneumococcal disease in young children in child care. Jama.

[46] Millar MR, Brown NM, Tobin GW, Murphy PJ, Windsor AC, Speller DC (1994). Outbreak of infection with penicillin-resistant Streptococcus pneumoniae in a hospital for the elderly. J Hosp Infect.

[47] Mandigers CM, Diepersloot RJ, Dessens M, Mol SJ, van Klingeren B (1994). A hospital outbreak of penicillin-resistant pneumococci in the Netherlands. Eur Respir J.

[48] Centers for Disease Control and Prevention. Hemorrhage and shock associated with invasive pneumococcal infection in healthy infants and children--New Mexico, 1993–1994. MMWR Morb Mortal Wkly Rep. 1995;43:949–952. 7865011

[49] Raymond J, Bingen E, Doit C, Brahimi N, Bergeret M, Badoual J, Gendrel D (1995). Failure of cefotaxime treatment in a patient with penicillin-resistant pneumococcal meningitis and confirmation of nosocomial spread by random amplified polymorphic DNA analysis. Clin Infect Dis.

[50] Gillespie SH, McHugh TD, Hughes JE, Dickens A, Kyi MS, Kelsey M (1997). An outbreak of penicillin resistant Streptococcus pneumoniae investigated by a polymerase chain reaction based genotyping method. J Clin Pathol.

[51] Fiore AE, Iverson C, Messmer T, Erdman D, Lett SM, Talkington DF, Anderson LJ, Fields B, Carlone GM, Breiman RF, Cetron MS (1998). Outbreak of pneumonia in a long-term care facility: antecedent human parainfluenza virus 1 infection may predispose to bacterial pneumonia. J Am Geriatr Soc.

[52] Nuorti JP, Butler JC, Crutcher JM, Guevara R, Welch D, Holder P, Elliott JA (1998). An outbreak of multidrug-resistant pneumococcal pneumonia and bacteremia among unvaccinated nursing home residents. N Engl J Med.

[53] Centers for Disease Control and Prevention. Outbreaks of pneumococcal pneumonia among unvaccinated residents of chronic-care facilities--Massachusetts, October 1995, Oklahoma, February, 1996, and Maryland, may-June 1996. MMWR Morb Mortal Wkly Rep. 1997;46:60–62. 9026712

[54] Musher DM, Groover JE, Reichler MR, Riedo FX, Schwartz B, Watson DA, Baughn RE, Breiman RF (1997). Emergence of antibody to capsular polysaccharides of Streptococcus pneumoniae during outbreaks of pneumonia: association with nasopharyngeal colonization. Clin Infect Dis.

[55] Sheppard DC, Bartlett KA, Lampiris HW (1998). Streptococcus pneumoniae transmission in chronic-care facilities: description of an outbreak and review of management strategies. Infect Control Hosp Epidemiol.

[56] Razzaq N, Riordan T, McNinch AW, Daneshmend TK (1998). A possible secondary case of pneumococcal meningitis. J Inf Secur.

[57] Craig AS, Erwin PC, Schaffner W, Elliott JA, Moore WL, Ussery XT, Patterson L, Dake AD, Hannah SG, Butler JC (1999). Carriage of multidrug-resistant Streptococcus pneumoniae and impact of chemoprophylaxis during an outbreak of meningitis at a day care center. Clin Infect Dis.

[58] de Galan BE, van Tilburg PM, Sluijter M, Mol SJ, de Groot R, Hermans PW, Jansz AR (1999). Hospital-related outbreak of infection with multidrug-resistant Streptococcus pneumoniae in the Netherlands. J Hosp Infect.

[59] Kellner JD, Gibb AP, Zhang J, Rabin HR (1999). Household transmission of Streptococcus pneumoniae, Alberta, Canada. Emerg Infect Dis.

[60] Leggiadro RJ, Schaberg DR (1999). Nosocomial pneumococcal infection: an outbreak. Hosp Pract (1995).

[61] Dagan R, Gradstein S, Belmaker I, Porat N, Siton Y, Weber G, Janco J, Yagupsky P (2000). An outbreak of Streptococcus pneumoniae serotype 1 in a closed community in southern Israel. Clin Infect Dis.

[62] Centers for Disease Control and Prevention. Outbreak of pneumococcal pneumonia among unvaccinated residents of a nursing home--New Jersey, April 2001. MMWR Morb Mortal Wkly Rep. 2001;50:707–710. 11787578

[63] Tan CG, Ostrawski S, Bresnitz EA (2003). A preventable outbreak of pneumococcal pneumonia among unvaccinated nursing home residents in New Jersey during 2001. Infect Control Hosp Epidemiol.

[64] Weiss K, Restieri C, Gauthier R, Laverdiere M, McGeer A, Davidson RJ, Kilburn L, Bast DJ, de Azavedo J, Low DE (2001). A nosocomial outbreak of fluoroquinolone-resistant Streptococcus pneumoniae. Clin Infect Dis.

[65] Melamed R, Greenberg D, Landau D, Khvatskin S, Shany E, Dagan R (2002). Neonatal nosocomial pneumococcal infections acquired by patient-to-patient transmission. Scand J Infect Dis.

[66] Crum NF, Wallace MR, Lamb CR, Conlin AM, Amundson DE, Olson PE, Ryan MA, Robinson TJ, Gray GC, Earhart KC (2003). Halting a pneumococcal pneumonia outbreak among United States marine corps trainees. Am J Prev Med.

[67] Subramanian D, Sandoe JA, Keer V, Wilcox MH (2003). Rapid spread of penicillin-resistant Streptococcus pneumoniae among high-risk hospital inpatients and the role of molecular typing in outbreak confirmation. J Hosp Infect.

[68] Sanchez JL, Craig SC, Kolavic S, Hastings D, Alsip BJ, Gray GC, Hudspeth MK, Ryan MA (2003). An outbreak of pneumococcal pneumonia among military personnel at high risk: control by low-dose azithromycin postexposure chemoprophylaxis. Mil Med.

[69] Banerjee A, Kalghatgi AT, Saiprasad GS, Nagendra A, Panda BN, Dham SK, Mahen A, Menon KD, Khan MA (2005). Outbreak of pneumococcal pneumonia among military recruits. Med J Armed Forces India.

[70] Carter RJ, Sorenson G, Heffernan R, Kiehlbauch JA, Kornblum JS, Leggiadro RJ, Nixon LJ, Wertheim WA, Whitney CG, Layton M (2005). Failure to control an outbreak of multidrug-resistant Streptococcus pneumoniae in a long-term-care facility: emergence and ongoing transmission of a fluoroquinolone-resistant strain. Infect Control Hosp Epidemiol.

[71] Centers for Disease Control and Prevention. Outbreak of invasive pneumococcal disease--Alaska, 2003–2004. MMWR Morb Mortal Wkly Rep. 2005;54:72–75. 15674187

[72] Zulz T, Wenger JD, Rudolph K, Robinson DA, Rakov AV, Bruden D, Singleton RJ, Bruce MG, Hennessy TW (2013). Molecular characterization of Streptococcus pneumoniae serotype 12F isolates associated with rural community outbreaks in Alaska. J Clin Microbiol.

[73] Birtles A, McCarthy N, Sheppard CL, Rutter H, Guiver M, Haworth E, George RC (2005). Multilocus sequence typing directly on DNA from clinical samples and a cultured isolate to investigate linked fatal pneumococcal disease in residents of a shelter for homeless men. J Clin Microbiol.

[74] Hansmann Y, Doyle A, Remy V, Jaulhac B, Christmann D, Lesens O, Perrocheau A (2006). An outbreak of pneumococcal pneumonia among residents of a retirement home in France during October 2003. Infect Control Hosp Epidemiol.

[75] Singh P, Jaiswal A, Handa S, Bhalwar R, Wankhede V, Banerjee A, Bhatnagar D, Kumar H (2006). Outbreak of pneumococcal pneumonia in Military Barracks. Indian J Community Med.

[76] Cashman P, Massey P, Durrheim D, Islam F, Merritt T, Eastwood K (2007). Pneumonia cluster in a boarding school--implications for influenza control. Commun Dis Intell Q Rep.

[77] Sheppard CL, Salmon JE, Harrison TG, Lyons M, George RC (2008). The clinical and public health value of non-culture methods in the investigation of a cluster of unexplained pneumonia cases. Epidemiol Infect.

[78] Romney MG, Hull MW, Gustafson R, Sandhu J, Champagne S, Wong T, Nematallah A, Forsting S, Daly P (2008). Large community outbreak of Streptococcus pneumoniae serotype 5 invasive infection in an impoverished, urban population. Clin Infect Dis.

[79] Vainio A, Lyytikainen O, Sihvonen R, Kaijalainen T, Teirila L, Rantala M, Lehtinen P, Ruuska P, Virolainen A (2009). An outbreak of pneumonia associated with S. pneumoniae at a military training facility in Finland in 2006. Apmis.

[80] Gupta A, Khaw FM, Stokle EL, George RC, Pebody R, Stansfield RE, Sheppard CL, Slack M, Gorton R, Spencer DA (2008). Outbreak of Streptococcus pneumoniae serotype 1 pneumonia in a United Kingdom school. Bmj.

[81] Mehiri-Zghal E, Decousser JW, Mahjoubi W, Essalah L, El Marzouk N, Ghariani A, Allouch P, Slim-Saidi NL (2010). Molecular epidemiology of a Streptococcus pneumoniae serotype 1 outbreak in a Tunisian jail. Diagn Microbiol Infect Dis.

[82] Balicer RD, Zarka S, Levine H, Klement E, Sela T, Porat N, Ash N, Dagan R (2010). Control of Streptococcus pneumoniae serotype 5 epidemic of severe pneumonia among young army recruits by mass antibiotic treatment and vaccination. Vaccine.

[83] Pichon B, Moyce L, Sheppard C, Slack M, Turbitt D, Pebody R, Spencer DA, Edwards J, Krahe D, George R (2010). Molecular typing of pneumococci for investigation of linked cases of invasive pneumococcal disease. J Clin Microbiol.

[84] Dawood FS, Ambrose JF, Russell BP, Hawksworth AW, Winchell JM, Glass N, Thurman K, Soltis MA, McDonough E, Warner AK (2011). Outbreak of pneumonia in the setting of fatal pneumococcal meningitis among US Army trainees: potential role of chlamydia pneumoniae infection. BMC Infect Dis.

[85] Vanderkooi OG, Church DL, MacDonald J, Zucol F, Kellner JD (2011). Community-based outbreaks in vulnerable populations of invasive infections caused by Streptococcus pneumoniae serotypes 5 and 8 in Calgary, Canada. PLoS One.

[86] Skoczynska A, Sadowy E, Krawiecka D, Czajkowska-Malinowska M, Ciesielska A, Przybylski G, Zebracka R, Hryniewicz W (2012). Nosocomial outbreak of Streptococcus pneumoniae Spain9VST15614 clone in a pulmonary diseases ward. Pol Arch Med Wewn.

[87] Fleming-Dutra K, Mbaeyi C, Link-Gelles R, Alexander N, Guh A, Forbes E, Beall B, Winchell JM, Carvalho Mda G, Pimenta F (2012). Streptococcus pneumoniae serotype 15A in psychiatric unit, Rhode Island, USA, 2010-2011. Emerg Infect Dis.

[88] Centers for Disease Control and Prevention. Notes from the field: Outbreak of severe respiratory illness in an assisted-living facility--Colorado, 2012. MMWR Morb Mortal Wkly Rep. 2013;62:230–1. PMC4605005 23535690

[89] Kuroki T, Ishida M, Suzuki M, Furukawa I, Ohya H, Watanabe Y, Konnai M, Aihara Y, Chang B, Ariyoshi K (2014). Outbreak of Streptococcus pneumoniae serotype 3 pneumonia in extremely elderly people in a nursing home unit in Kanagawa, Japan, 2013. J Am Geriatr Soc.

[90] Ben-David D, Schwaber MJ, Adler A, Masarwa S, Edgar R, Navon-Venezia S, Schwartz D, Porat N, Kotlovsky T, Polivkin N (2014). Persistence and complex evolution of fluoroquinolone-resistant Streptococcus pneumoniae clone. Emerg Infect Dis.

[91] Schillberg E, Isaac M, Deng X, Peirano G, Wylie JL, Van Caeseele P, Pillai DR, Sinnock H, Mahmud SM (2014). Outbreak of invasive Streptococcus pneumoniae serotype 12F among a marginalized inner-city population in Winnipeg, Canada, 2009-2011. Clin Infect Dis.

[92] Suryam V, Bhatti VK, Kulkarni A, Mahen A, Nair V (2015). Outbreak control of community acquired pneumonia in a large military training institution. Med J Armed Forces India.

[93] Thomas HL, Gajraj R, Slack MP, Sheppard C, Hawkey P, Gossain S, Drew CM, Pebody RG (2015). An explosive outbreak of Streptococcus pneumoniae serotype-8 infection in a highly vaccinated residential care home, England, summer 2012. Epidemiol Infect.

[94] Kunwar R, Sidana N (2015). Mass chemoprophylaxis in control of pneumococcal pneumonia outbreak in a military training Centre. Indian J Public Health.

[95] Sheppard CL, Clark J, Slack MP, Fry NK, Harrison TG (2016). Use of a serotype-specific urine immunoassay to determine the course of a hospital outbreak of Streptococcus pneumoniae complicated by influenza a. JMM Case Rep.

[96] Ewing J, Patterson L, Irvine N, Doherty L, Loughrey A, Kidney J, Sheppard C, Kapatai G, Fry NK, Ramsay M, Jessop L (2017). Serious pneumococcal disease outbreak in men exposed to metal fume - detection, response and future prevention through pneumococcal vaccination. Vaccine.

[97] Jauneikaite E, Khan-Orakzai Z, Kapatai G, Bloch S, Singleton J, Atkin S, Shah V, Hatcher J, Samarasinghe D, Sheppard C (2017). Nosocomial outbreak of drug-resistant Streptococcus pneumoniae serotype 9V in an adult respiratory medicine Ward. J Clin Microbiol.

[98] Shayegani M, Parsons LM, Gibbons WE, Campbell D (1982). Characterization of nontypable Streptococcus pneumoniae-like organisms isolated from outbreaks of conjunctivitis. J Clin Microbiol.

[99] Ertugrul N, Rodriguez-Barradas MC, Musher DM, Ryan MA, Agin CS, Murphy SJ, Shayegani M, Watson DA (1997). BOX-polymerase chain reaction-based DNA analysis of nonserotypeable Streptococcus pneumoniae implicated in outbreaks of conjunctivitis. J Infect Dis.

[100] Centers for Disease Control and Prevention. Pneumococcal conjunctivitis at an elementary school--Maine, September 20–December 6, 2002. MMWR Morb Mortal Wkly Rep. 2003;52:64–66. 12578323

[101] Martin M, Turco JH, Zegans ME, Facklam RR, Sodha S, Elliott JA, Pryor JH, Beall B, Erdman DD, Baumgartner YY (2003). An outbreak of conjunctivitis due to atypical Streptococcus pneumoniae. N Engl J Med.

[102] Zegans ME, Sanchez PA, Likosky DS, Allar RT, Martin M, Schwartzman JD, Pryor JH, Turco JH, Whitney CG (2009). Clinical features, outcomes, and costs of a conjunctivitis outbreak caused by the ST448 strain of Streptococcus pneumoniae. Cornea.

[103] Crum NF, Barrozo CP, Chapman FA, Ryan MA, Russell KL (2004). An outbreak of conjunctivitis due to a novel unencapsulated Streptococcus pneumoniae among military trainees. Clin Infect Dis.

[104] Buck JM, Lexau C, Shapiro M, Glennen A, Boxrud DJ, Koziol B, Whitney CG, Beall B, Danila R, Lynfield R (2006). A community outbreak of conjunctivitis caused by nontypeable Streptococcus pneumoniae in Minnesota. Pediatr Infect Dis J.

[105] Hennink M, Abbas Z, McDonald RR, Nagle E, Montgomery KL, Diener T, Horsman GB, Levett PN (2006). Streptococcus pneumoniae outbreak in a rural Regina community. Can Commun Dis Rep.

[106] Marton A, Nagy A, Katona G, Fekete F, Votisky P, Lajos Z (1997). Nosocomial Streptococcus pneumoniae infection causing children's acute otitis media. Int J Antimicrob Agents.

[107] Nakashima T, Fukushima K, Tahara M, Sugata KI, Ogawa T, Sugata A, Gunduz M, Ueki Y, Uno Y, Nishizaki K (2001). Random amplified polymorphic DNA analysis applied to acute otitis media caused by penicillin non-susceptible Streptococcus pneumoniae. J Infect Chemother.

[108] Guillet M, Zahar JR, Timsit MO, Grandin L, Carbonnelle E, Join-Lambert O, Quesne G, Nassif X, Mejean A, Carbonne A (2012). Horizontal transmission of Streptococcus pneumoniae in the surgical ward: a rare source of nosocomial wound infection. Am J Infect Control.

[109] Gilman BB, Anderson GW (1938). A community outbreak of type I pneumococcus infection. Am J Hyg.

[110] Centers for Disease Control and Prevention. From the Centers for Disease Control and Prevention. Outbreaks of pneumococcal pneumonia among unvaccinated residents in chronic-care facilities--Massachusetts, October 1995, Oklahoma, February 1996, and Maryland, may-June 1996. Jama. 1997;277:452–453. 9020259

[111] Centers for Disease Control and Prevention. Heymann, DL. Control of Communicable Diseases Manual. 20th edn. Washington, DC: American Public Health Association; 2015.

[112] Siegel JD, Rhinehart E, Jackson M, Chiarello L (2007). Guideline for isolation precautions: preventing transmission of infectious agents in health care settings. Am J Infect Control.

[113] Kimberlin D, Brady M, Jackson M, Long S, Pediatrics AAo (2015). In Red Book: 2015 Report of the Committee on Infectious Diseases.

[114] Grabenstein JD, Musey LK (2014). Differences in serious clinical outcomes of infection caused by specific pneumococcal serotypes among adults. Vaccine.

[115] Centers for Disease Control and Prevention. Use of 13-valent pneumococcal conjugate vaccine and 23-valent pneumococcal polysaccharide vaccine for adults with immunocompromising conditions: recommendations of the Advisory Committee on Immunization Practices (ACIP). MMWR Morb Mortal Wkly Rep. 2012;61:816–9. 23051612

[116] Tomczyk S, Bennett NM, Stoecker C, Gierke R, Moore MR, Whitney CG, Hadler S, Pilishvili T (2014). Use of 13-valent pneumococcal conjugate vaccine and 23-valent pneumococcal polysaccharide vaccine among adults aged >/=65 years: recommendations of the advisory committee on immunization practices (ACIP). MMWR Morb Mortal Wkly Rep.

[117] Bridges CB, Woods L, Coyne-Beasley T (2013). Advisory committee on immunization practices (ACIP) recommended immunization schedule for adults aged 19 years and older--United States, 2013. MMWR Suppl.

[118] Prevention CfDCa: Recommended Immunization Schedule for Adults Aged 19 Years or Older, United States, 2018**.** (Services UDoHaH ed.; 2018. 10.7326/M17-3439 29404596

[119] Frenck RW, Fiquet A, Gurtman A, van Cleeff M, Davis M, Rubino J, Smith W, Sundaraiyer V, Sidhu M, Emini EA (2016). Immunogenicity and safety of a second administration of 13-valent pneumococcal conjugate vaccine 5 years after initial vaccination in adults 50 years and older. Vaccine.

[120] Remschmidt C, Harder T, Wichmann O, Bogdan C, Falkenhorst G (2016). Effectiveness, immunogenicity and safety of 23-valent pneumococcal polysaccharide vaccine revaccinations in the elderly: a systematic review. BMC Infect Dis.

[121] Grabenstein JD, Manoff SB (2012). Pneumococcal polysaccharide 23-valent vaccine: long-term persistence of circulating antibody and immunogenicity and safety after revaccination in adults. Vaccine.

[122] Mosser JF, Grant LR, Millar EV, Weatherholtz RC, Jackson DM, Beall B, Craig MJ, Reid R, Santosham M, O'Brien KL (2014). Nasopharyngeal carriage and transmission of Streptococcus pneumoniae in American Indian households after a decade of pneumococcal conjugate vaccine use. PLoS One.

[123] Pessoa D, Hoti F, Syrjanen R, Sa-Leao R, Kaijalainen T, Gomes MG, Auranen K (2013). Comparative analysis of Streptococcus pneumoniae transmission in Portuguese and Finnish day-care centres. BMC Infect Dis.

[124] Hussain M, Melegaro A, Pebody RG, George R, Edmunds WJ, Talukdar R, Martin SA, Efstratiou A, Miller E (2005). A longitudinal household study of Streptococcus pneumoniae nasopharyngeal carriage in a UK setting. Epidemiol Infect.

[125] Shimada J, Yamanaka N, Hotomi M, Suzumoto M, Sakai A, Ubukata K, Mitsuda T, Yokota S, Faden H (2002). Household transmission of Streptococcus pneumoniae among siblings with acute otitis media. J Clin Microbiol.

[126] Edouard S, Al-Tawfiq JA, Memish ZA, Yezli S, Gautret P. Impact of the hajj on pneumococcal carriage and the effect of various pneumococcal vaccines. Vaccine. 2017. 10.1016/j.vaccine.2018.09.017. 10.1016/j.vaccine.2018.09.017 30236632

[127] Davis BM, Aiello AE, Dawid S, Rohani P, Shrestha S, Foxman B (2012). Influenza and community-acquired pneumonia interactions: the impact of order and time of infection on population patterns. Am J Epidemiol.

[128] Rudd JM, Ashar HK, Chow VTK, Teluguakula N: Lethal synergism between influenza and Streptococcus pneumoniae**.** J Infect Pulm Dis 2016, 2**:**10.16966/12470-13176.16114. 10.16966/2470-3176.114 PMC5154682 27981251

[129] Launes C, de-Sevilla MF, Selva L, Garcia-Garcia JJ, Pallares R, Munoz-Almagro C (2012). Viral coinfection in children less than five years old with invasive pneumococcal disease. Pediatr Infect Dis J.

[130] Wolter N, Tempia S, Cohen C, Madhi SA, Venter M, Moyes J, Walaza S, Malope-Kgokong B, Groome M, du Plessis M (2014). High nasopharyngeal pneumococcal density, increased by viral coinfection, is associated with invasive pneumococcal pneumonia. J Infect Dis.

[131] CDC: Interim guidance for use of 23-valent pneumococcal polysaccharide vaccine during novel influenza A (H1N1) outbreak. http://www.cdc.gov/h1n1flu/guidance/ppsv_h1n1.htm 2009.

[132] Gupta RK, George R, Nguyen-Van-Tam JS (2008). Bacterial pneumonia and pandemic influenza planning. Emerg Infect Dis.

[133] McCullers JA (2006). Insights into the interaction between influenza virus and pneumococcus. Clin Microbiol Rev.

[134] ABCs Report: Streptococcus pneumoniae, 2015. (Prevention CfDCa ed.; 2016.

[135] Dagan R, Klugman KP (2008). Impact of conjugate pneumococcal vaccines on antibiotic resistance. Lancet Infect Dis.

[136] Tomczyk S, Arriola CS, Beall B, Benitez A, Benoit SR, Berman L, Bresee J, da Gloria CM, Cohn A, Cross K (2016). Multistate outbreak of respiratory infections among unaccompanied children, June 2014–July 2014. Clin Infect Dis.

