# Supplementary figures and images for: Streptococcus pneumoniae outbreaks and implications for transmission and control: a systematic review
Source: Pneumonia (Nathan). 2018 Nov 5;10:11. doi: 10.1186/s41479-018-0055-4 (PMC6217781; doi:10.1186/s41479-018-0055-4)

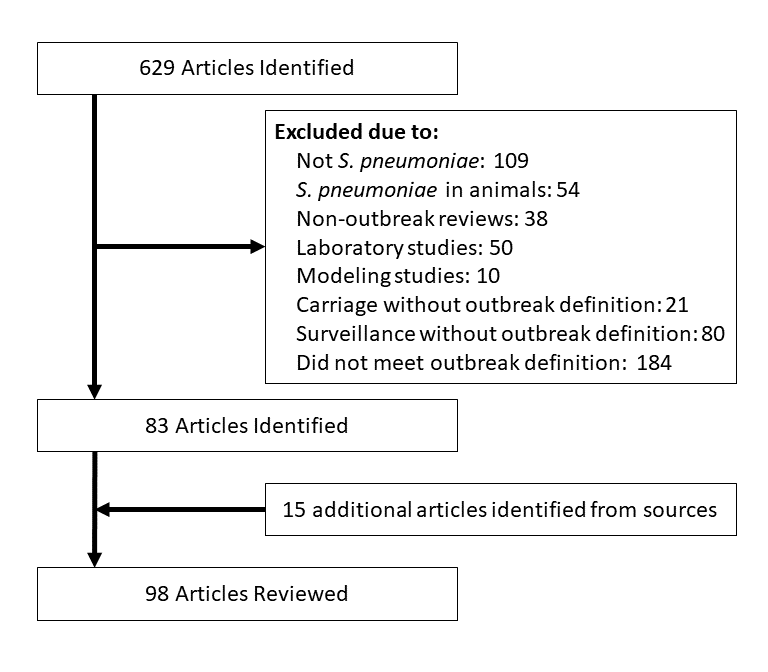

Supplement: Supplementary file 1 — Figure S1. Article exclusion flow diagram. (PNG 22 kb) [file 41479_2018_55_MOESM1_ESM.png]
